# Assessment of cheese frauds, and relevant detection methods: A systematic review

**DOI:** 10.1016/j.fochx.2023.100825

**Published:** 2023-08-06

**Authors:** Amirhossein Abedini, Mahla Salimi, Yeganeh Mazaheri, Parisa Sadighara, Mahmood Alizadeh Sani, Elham Assadpour, Seid Mahdi Jafari

**Affiliations:** a Students Scientific Research Center (SSRC), Tehran University of Medical Sciences, Tehran, Iran; b Division of Food Safety and Hygiene, Department of Environmental Health, School of Public Health, Tehran University of Medical Sciences, Tehran, Iran; c Student Research Committee, Department of Food Science and Technology, National Nutrition and Food Technology Research Institute, Faculty of Nutrition Science and Food Technology, Shahid Beheshti University of Medical Sciences, Tehran, Iran; d Food Industry Research Co., Gorgan, Iran; e Food and Bio-Nanotech International Research Center (Fabiano), Gorgan University of Agricultural Sciences and Natural Resources, Gorgan, Iran; f Department of Food Materials and Process Design Engineering, Gorgan University of Agricultural Sciences and Natural Resources, Gorgan, Iran

**Keywords:** Adulterants, Cheese, Dairy products, Food safety, Frauds

## Abstract

Dairy products are widely consumed in the world due to their nutritional and functional characteristics. This group of food products are consumed by all age groups due to their health-giving properties. One of these products is cheese which has a high price compared to other dairy products. Because of this, it can be prone to fraud all over the world. Fraud in food products threatens the world's food safety and can cause serious damage to human health. There are many concerns among food authorities in the world about the fraud of food products. FDA, WHO, and the European Commission provide different legislations and definitions for fraud. The purpose of this review is to identify the most susceptible cheese type for fraud and effective methods for evaluating fraud in all types of cheeses. For this, we examined the Web of Science, Scopus, PubMed, and ScienceDirect databases. Mozzarella cheese had the largest share among all cheeses in terms of adulteration due to its many uses. Also, the methods used to evaluate different types of cheese frauds were PCR, Spectrometry, stable isotope, image analysis, electrophoretic, ELISA, sensors, sensory analysis, near-infrared and NMR. The methods that were most used in detecting fraud were PCR and spectrometry methods. Also, the least used method was sensory evaluation.

## Highlights

• This article reviews the assessment of cheese frauds and related detection methods.
• Mozzarella cheese had the largest share among all cheeses in terms of adulteration.
• The PCR and spectrometry methods were most used in detecting fraud of cheeses.
• The least used method was sensory evaluation.

## 1 Introduction

**Fig. 1:**
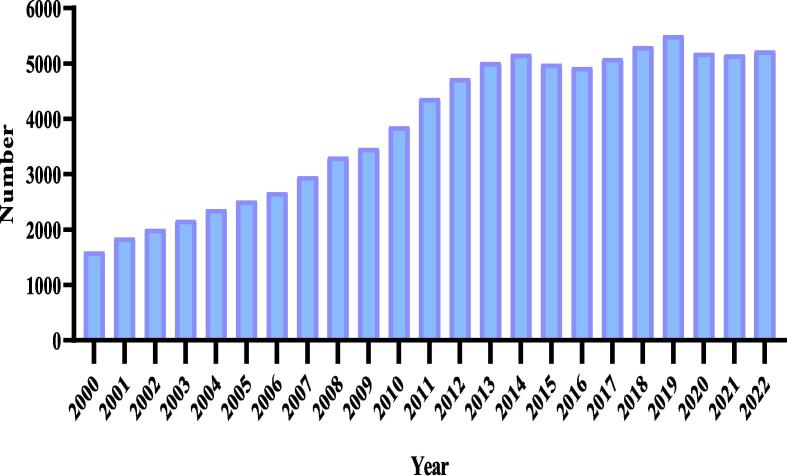
Publication reports by scientific databases about food product fraud.

In recent years, food fraud (FF) has increased due to the spread of poverty and economic problems (Handford et al., 2016, Spink et al., 2019) (Fig. 1). Soon and Wahab (2020) estimated the impact of fraud on the food industry to be >$50 billion annually (Soon & Wahab, 2022). Montgomery, Haughey, and Elliott (2020) reported that cheese had the highest adulteration rate among dairy products. They observed that the replacement of ingredients and reduced fat in cheeses is one of the important factors in fraud. In the analysis of salt content, 30 dairy products were adulterated in 29 cases of cheese. Also, among the 15 reviewed dairy products, 10 of the cheeses had less fat than claimed (Montgomery et al., 2020). In another study, Owolabi et al. (2021) observed that according to the Food Fraud Database, dairy products had the highest fraud rate among food products (Owolabi & Olayinka, 2021). Regulatory agencies spend a lot of money every year to assess and reduce FF because it has a fundamental connection to nutrition and public health (Spink et al., 2019, Bouzembrak et al., 2018).

**Fig. 2:**
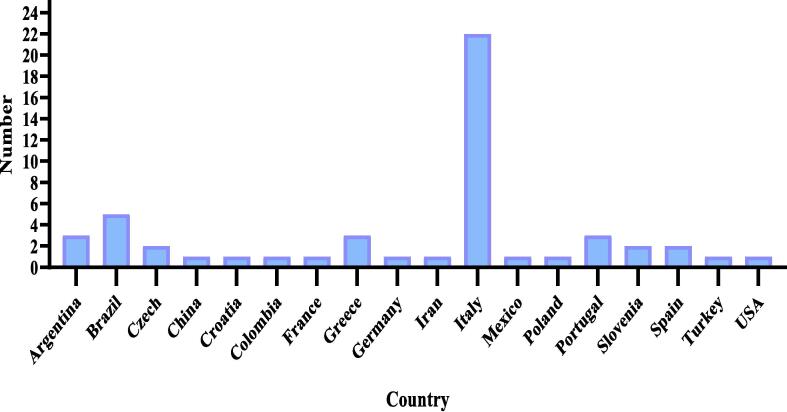
The number of publications from different countries included in the systematic study.

Monitoring food products, especially high-consumption products such as cereals and dairy products, is more important (Sharma & Kaushal, 2021). It should be noted that approximately 25 to 30 % of the human diet is devoted to dairy products such as milk, butter, cheese, ice cream, and cream. But the consumption of dairy products is different in countries around the world; for example, Asian countries tend to consume fewer dairy products, and in Europe, the consumption of dairy products is high (Stobiecka, Król, & Brodziak, 2022) (Fig. 2). Studies show that the market for the sale and consumption of dairy products will expand until 2025, so that 2.0 % for non-fat dry milk and 2.1 % for whole milk, butter, and cheese will increase at an annual rate of 1.7 % and 1.4 %, respectively (Valdés, Beltrán, Mellinas, Jiménez, & Garrigós, 2018).

Also, statistics show that milk production will reach 981 million tons by 2028. In addition, the expansion of cattle farms (1.2 % p.a.) will be higher than expected (0.4 %). In addition, FAO reports that the main milk producers, Pakistan and India, will have more than 30 % of the global production share in 2028 (OECD and FAO, 2019). One of the important cases will be the consumption of fresh milk in the coming years. It seems that the consumption of fresh milk will increase with the increase in consumer information and market demand. Meanwhile, the methods of fraud investigation will be much more important because the speed and accuracy of these methods must be constantly improved to match the market demand (Sadighara, Abedini, Mahvi, et al., 2023). World per capita consumption of fresh dairy products is projected to increase by 1.0 % p.a (OECD and FAO, 2019).

In terms of milk solids per capita, the consumption of dairy products will be different in different regions of the world. So income will be one of the most determining factors in the consumption of these products. In Europe and North America, overall per capita demand for fresh dairy products is declining, but the composition of demand has been shifting over the last several years towards dairy fat, e.g., full-fat drinking milk and cream. This is to some extent due to recent studies that have shed a more positive light on the health benefits of dairy fat consumption (OECD and FAO, 2019).

As well as to growing consumer preference for taste and less processed foods. Cheese consumption, the second most important dairy product in terms of milk solids, occurs primarily in Europe, North America, and Oceania, and per capita consumption is expected to continue to increase.

It should be noted that buying and selling milk (fresh and product) on a large scale in liquid form is economically expensive, and it seems that the import and export of milk powder will increase significantly in developing countries in the coming years (OECD and FAO, 2019).

Perishability and high water content are among the factors influencing milk trade in the world. It is estimated that only 8 % of the milk produced in the world is traded internationally. But countries like China have significantly increased the import of milk. The four major exporters of dairy products in the base period are New Zealand, the European Union, the United States, and Australia. These four counties are expected to jointly account for around 75 % of cheese, 78 % of WMP, 79 % of butter, and 81 % of SMP exports in 2028. The European Union (around 48 %), United States, and New Zealand will have the highest cheese exports in the world by 2028. The United Kingdom, the Russian Federation, Japan, the European Union, and China are projected to be the top five cheese importers in 2028 (OECD and FAO, 2019).

The economic debate is also an important aspect to note, as most studies have shown that the harmful substance has not been used in fraud, but in the production of cheese, various kinds of milk or other additives have been used (Abedini et al., 2022, Sadighara et al., 2023). This information shows that economic problems in the food industry are aspects that should be considered more than before because the economy is a major motivator for FF (Moyer et al., 2017, Meerza and Gustafson, 2019, Abedini et al., 2021). Economically motivated adulteration (EMA) happens when someone purposefully omits, removes, or replaces a valuable component of a meal. Adding anything to food to make it seem better or more valuable is another instance of EMA. For instance, producers deceive consumers when they mix less costly vegetable oils with expensive olive oil while advertising the product as 100 % olive oil (US Food and Drug Administration, 2016). The Food and Drug Administration (FDA) frequently deals with FF, but EMA can also involve other goods, such as cosmetics and animal feeds. A few EMA kinds also violate brand integrity. Because FF is intended to go undetected, it can be challenging to determine how frequently it occurs or how much of an economic impact it has. According to estimates by experts, FF affects 1 % of the global food business and costs between $10 and $15 billion annually; however, more recent estimates by experts put the cost as high as $40 billion (Fraud, 2023).

The methods that can be used to detect fraud in cheese products are stable isotope, silicon photonic immune sensor, image analysis, Iso Electro Focusing (IEF), liquid chromatography-tandem mass spectrometry (LC-MS), nano flow RP LC-MS (RP-LC–MS), polymerase chain reaction (PCR), droplet digital PCR (ddPCR), Fourier transform near-infrared (FT-NIR) spectroscopy, competitive ELISA, QPCR ASSAY method, sensory analysis, electrophoresis (SDS-PAGE), LC/electrospray ionization/MS (LC/ESI-MS), differential scanning calorimetry (DSC) coupled with cluster analysis, and FTIR-ATR spectroscopy coupled with multivariate and NIR spectroscopy (Hong et al., 2017, Visconti et al., 2020, Cardoso et al., 2019, Mininni et al., 2009, Haddi et al., 2010). For example, in 2022, Kritikou et al. used the Matrix-assisted Laser Desorption/Ionization-Time-Of-Flight MS method (MALDI-TOF-MS) to evaluate cow's milk in feta cheese. They found a way to use biomarkers to detect cow's milk, even if it's only 1 % of the milk (Kritikou et al., 2022). Another study by Caira et al. in 2019 evaluated mozzarella cheese fraud. For this purpose, they used the IEF method as an alternative to conventional methods such as mass spectrometry. Thus, combined proteomic methods were here integrated with optimized western blotting protocols in solving the complex IEF pattern of casein (CN) mixtures observed when Italian and foreign WB milk are mixed. Identification of internally deleted αs1-CN hepta-phosphorylated species, as well as of still unknown β-CN A *hexa*-phosphorylated and *N*-terminally-nicked β-CN A phosphorylated forms present uniquely in foreign water buffalo milk samples, allowed recognizing these molecules as adulteration markers. IEF could detect amounts of adulteration as low as 3 % v/v (Caira et al., 2019).

The purpose of this article is to answer the question: what is the rate of fraud in cheeses (Parmesan, Mozzarella, Hard, Soft, Sheep, Ricotta, Goat, Caprine, Feta, Parmigiano Reggiano, Halloumi, Stelvio and Serra da Estrela), what are the ingredients used to adulterate cheese and which method of fraud assessment is most used for different types of cheese.

### 1.1 Defining food fraud

FF can be investigated in different terms. One of the definitions reports that the purposeful and intentional replacement, addition, tampering, or misrepresentation of food, food components, or food packaging, as well as the making of false or deceptive assertions about a product, are together referred to as “food fraud” (Spink & Moyer, 2011). Other definitions are the deliberate adulteration of food for commercial gain (Everstine et al., 2013, Spink et al., 2015); an intentional deception motivated by the possibility of financial gain (Charlebois, Schwab, Henn, & Huck, 2016); a criminal deception for financial advantage using food, including the subcategory that FDA defines as EMA (Moyer et al., 2017); A change made to food on purpose that could deceive a person who eats it and cause them to lose money (Cruse, 2019); using food to commit illegal fraud for financial gain (Spink, Bedard, et al. 2019). Another definition that examines FF more completely is the definition of Ellis et al. (2015), they reported that any act committed when food is knowingly marketed for commercial advantage with the intention of misleading customers, is referred to as EMA in the USA and infrequently overseas.

**Fig. 3:**
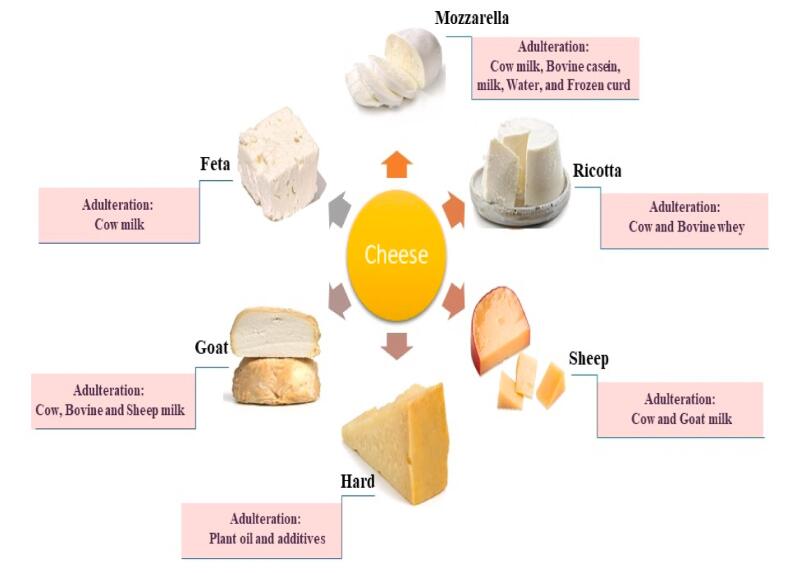
Adulteration in some types of cheese.

Trading in food that is unsafe for ingestion or dangerous, or purposefully mislabeling food, are two of the primary categories. The latter may contain exaggerated claims about the geographical origin of the product, the components, a lower-quality replacement (such as myrtle in place of oregano), or perhaps even harmful elements that are not meant for human consumption (i.e. industrial dyes). When adulteration is done on purpose, the phrases “food fraud” and “food adulteration” have the same meaning (Ellis, Muhamadali, Haughey, Elliott, & Goodacre, 2015) (Fig. 3). In Europe, FF refers to situations in which a deliberate breach of the European Food Safety Authority (EFSA) is made to deceive consumers for financial or economic advantage (Akbari-Adergani et al., 2022).

Fake food can occur when people lie about the quality of food products, such as their purity and safety. A fake product's expiry date and make sure the steps to make it are done correctly. Changing the direction of products from where they were supposed to go or sold). Being honest and ethical as a person (for example, not lying or cheating). It also talks about making sure information is true and correct. The information about the food might not be right if there are mistakes, old information, or lies, or if important documents like health certificates are missing (Bouzembrak et al., 2018, Koubová et al., 2018, Robson et al., 2021).

### 1.2 Formulation parameters and cheese production process

Cheese formulas often rely on the kind of process of cheese being produced as well as the kinds of end-uses that processed cheese will be intended for. The ultimate functional qualities of processed cheese are influenced by a range of chemical and compositional factors in different ways (Kapoor & Metzger, 2008). The cost, availability of other ingredients, the kind and age of natural cheese, and whether or not rework is present vary from day to day (Talbot-Walsh, Kannar, & Selomulya, 2018). These are some limitations that producers must overcome while creating their processed cheese to produce a finished good with reliable functional characteristics every day. As a result, choosing the components for the same processed cheese recipe involves different permutations and combinations processed cheese producers have traditionally chosen the ingredient combination for a particular formula based on their experience. They are now using computer programs to help make cheese. They can tell the program what they want in the cheese and it will find the cheapest way to make it.

As already mentioned, the functional qualities of processed cheese are influenced by a variety of chemical and compositional characteristics. These include the amount of fat, moisture, pH, calcium, lactose, intact casein, and whey proteins (Pelayo et al., 2021, Tadjine et al., 2019). It should be noted that the characteristics of the produced cheese are not always the same, because the raw materials have different characteristics. Different amounts of calcium, pH, and casein in the original ingredients of natural cheese can create changes in the final processed cheese. These changes can affect how the cheese works in recipes (Ayvaz et al., 2021, Khorshidian et al., 2022). The kind and quantity of emulsifying salts added to alter the pH and the calcium status of processed cheese in addition to the inherent changes in cheese that impacts the total calcium content, pH, and intact casein content of the final processed cheese (Salek et al., 2019). The amount of whey proteins and lactose in processed cheese is affected by other ingredients. For example, milk with no fat, milk that has been dried, and a concentrated form of protein found in whey. Functional characteristics of processed cheese are greatly impacted by differences in its chemical properties that occur throughout a processed cheese formation (Rocha & Guerra, 2020). The ultimate functional qualities of processed cheese are also influenced by the kind and quantity of rework added to the formulation. Also, to produce cheese with consistent functional qualities, it is crucial to manage the formulation parameters (Nogueira et al., 2018).

Cheese production includes various methods. Sometimes omitting one of the steps can be considered one of the signs of fraud. Because each stage of the cheese production process has a function that affects the characteristics of the final product. Mainly the steps include ingredient selection and preparation; natural cheese should be chosen and ground (based on age, pH, taste, and intact casein content), choosing the suitable emulsifying salt, formulation, and calculation of additional components (to achieve the desired moisture, fat, salt, and pH values of the finished product by legal requirements), processing and storing processed cheese (heating and combining during cooking), packaging, chilling, and storing (Kapoor and Metzger, 2008, Çelik and Yuksel, 2016, Sadighara et al., 2022).

## 2 Methods

This systematic review was written using the PRISMA checklist; according to the principles of the article, 2 different authors have reviewed all the steps of searching, quality assessment and inclusion and exclusion criteria, and data extraction to prevent bias.

### 2.1 Search strategy

Articles were searched in databases on January 21, 2022, and there was no limitation (1991–2022) on searching for them. The chosen databases were PubMed, Science direct, web of science, and Scopus. The keywords used in the systematic search included: (cheese or feta or mozzarella or ricotta or “goat cheese” or “sheep cheese”) and (Fraud or “cheese fraud” or “cheese adulteration”).

In the search of 4 main databases, we found 365 articles in this field. The first step in our work was to carefully study the title and abstract. Studies that did not meet the criteria were excluded from the article. Two authors (A. A and M.S) were responsible for the detailed review of the articles. At this stage, the full text of the studies was taken, and according to the table and protocol, information was received from them.

### 2.2 Inclusion and exclusion criteria

The two reviewers (A.A and M.S) searched the keywords in databases independently. Investigation of livestock age, geographical origin, non-English articles, reviews, chapters of the book, unrelated enzymatic activities, and articles that did not pay attention to fraud were excluded**.** The inclusion criteria for this systematic review included original articles that investigate cheese fraud or cheese adulteration. Articles that could confirm the inclusion criteria were fully reviewed.

### 2.3 Data extraction

Based on the method we designed in Table 1, several items, including the name of the first author, year of publication, country, type of cheese, type of fraud, and evaluation, including the study method and the result of the study, were written in Table 1.

**Table 1:** A summary of the studies related to the investigation of cheese fraud**.**

Country	Type of cheese	Type of Fraud	Fraud Assessment	Ref
Greece	Buffalo mozzarella and feta	Cow’s milk	A silicon photonic immune sensor was used for the first time for detecting bovine milk in buffalo cheeses. Bovine milk amount in feta and mozzarella cheese could be quantified as low as 0.25 % and 0.5 % (w/w).	(Angelopoulou et al., 2021)
Italy	Mozzarella di Bufala Campana (MBC)	Non-Mediterranean water buffalo (WB)	IEF methods were applied to observe patterns of casein in Italian and foreign WB milk mixtures. This method can detect amounts of adulteration as low as 3 % v/v.	(Caira et al., 2019)
Italy	Italian ricotta	Cow's whey	Nano flow RP-LC–MS/MS was applied to analyze peptide mixtures. It can detect bovine milk amounts as low as 0.5 % among buffalo, sheep, and goats.	(Camerini et al., 2016)
Italy	Goats’ milk products	Cow's milk	The assessment was based on specific whey protein presence. The minimum detectable amount of cow milk was 2 and 4 % in milk mixtures and cheeses, respectively.	(Cartoni, Coccioli, Jasionowska, & Masci, 1999)
Turkey	Sheep’s cheese	Adulteration	Samples were tested with the immune chromatographic method which could detect a 0.5 % adulteration of sheep’s cheese with cow’s milk. 52 % of cheese samples were found with no adulteration.	(Colak, Aydin, Nazli, & Ergun, 2006)
Italy	Buffalo Mozzarella	Bovine’s milk	ddPCR was developed for detecting the DNA of cow and/or buffalo milk in PDO buffalo mozzarella cheese. According to its high sensitivity, further tests need to be conducted to prove this technique for routine tests.	(Cutarelli et al., 2021)
Italy	Water Buffalo Mozzarellas	Cow's milk	LC-MS could detect cow milk in adulterated mozzarellas with the help of β-carotene and ergocalciferol quantification (detection limit of 5 % (w/w)). This method can be conveniently used for the authentication of Water Buffalo Mozzarellas.	(Bosco et al., 2018)
Poland	Hard cheese	Plant oil addition	According to their results, fat content can cause significant differences in fluorescent intensities. Fluorescence spectroscopy was applied for detecting cheese adulteration, combined with multiple linear regression models for calculating the level of adulteration.	(Dankowska, Małecka, & Kowalewski, 2015)
Italy	Dairy products	Adulteration	18 commercial dairy samples were analyzed by IEF and real-time PCR TaqMan. The 4 TaqMan method can detect fraud and mislabeling in milk and dairy products. 2 among 18 samples contained species not declared in the label	(Di Domenico et al., 2017)
Czech Republic	Goat cheeses	Cow’s milk	The techniques employed in detecting the adulteration of cow's milk included FT-NIR spectroscopy and competitive enzyme-linked immunosorbent assay (ELISA). Whilst the ELISA method was unable to provide a sufficient level of accuracy, the application of FT-NIR spectroscopy has demonstrated the capacity to detect cow's milk at concentrations as low as 1 % in goat cheese samples. Consequently, this method holds significant potential for effectively detecting cow's milk admixture in cheese products.	(Dvorak et al., 2016)
Italy	Italian Mozzarella	Bovine's milk	A PCR method was approached, which could determine bovine-specific mitochondrial DNA sequence in isolated DNA from cheese matrix. This method can detect amounts of bovine milk as low as 0.5 %.	(Feligini et al., 2005)
Italy	Water buffalo ricotta	Bovine's whey	The present study employed an isoelectric focusing technique to identify the presence of bovine whey in water buffalo Ricotta samples. The results demonstrated that bovine whey could be detected at concentrations exceeding 5 % (v/v).	(Fuselli et al., 2015)
–	Caprine	Bovine's milk	QPCR ASSAY method was successfully implemented.	(Fekete et al., 2017)
Brazil	Fresh goat cheese	Bovine's milk	A duplex PCR test was used to test goat cheeses for assessing adulteration. The test could detect 0.5 % (v/v) cow milk. All samples were identified with adulterated cow milk.	(Golinelli et al., 2014)
Brazil	Buffalo mozzarella	Cow's milk	The application of SDS-PAGE was employed to detect the inclusion of bovine milk. The proposed investigation involves a comparative analysis of electrophoretic profiles of proteins and peptides within cheese samples. The analysis conducted on a set of 18 commercial buffalo mozzarella samples, with a fat content of 28 %, revealed a proclivity for the inclusion of cow's milk as an additive. Moreover, they have demonstrated that the identification of cow's milk fortification cannot be achieved through the analysis of chemical and physicochemical characteristics.	(Gonçalves et al., 2017)
Italy	Goat’s and cow’s cheeses	Sheep's milk	A methodology utilizing LC/ESI-MS was developed to detect and determine the proportion of sheep's milk present in cheeses produced from cow's and goat's milk. This was achieved through the analysis of a peptide unique to sheep milk. The instrument is capable of measuring the concentration of sheep's milk in cheese samples up to a limit of 2 %.	(Guarino et al., 2010)
China	Goat milk and cheese	Cow's milk	A novel triplex TaqMan real-time PCR methodology has been established for the precise identification of the presence of cow milk in fraudulent practices. The identification of goat and cow DNA was achieved with a precision of 0.01 and 0.05 ng of DNA in milk and 327 cheese samples from cows, respectively. A similarly accurate identification was obtained from goat milk and cheese samples, with a precision of 0.005 and 0.01 ng of DNA, respectively.	(Guo et al., 2019)
Mexico	Cheese	Vegetable oil	Adulterated samples with a minimum amount of 5 g/L vegetable fat could be determined with DSC coupled with cluster analysis.	(Herman-Lara et al., 2017)
Slovenia	Caprine and ovine	Bovine's milk	A total of 17 samples of caprine cheese and 24 samples of ovine cheese were subjected to qPCR analysis to ascertain the presence of bovine, caprine, and ovine DNA. According to the data obtained, 17 % of the cheese samples analyzed demonstrated a concentration of ovine milk exceeding 5 % whereas 22 % of the samples showed the detectability of ovine milk lower than or equal to 5 %.	(Klančnik et al., 2016)
Greece	PDO feta	Cow's milk	MALDI-TOF-MS could detect the amount of cow milk down to 1 % with the help of biomarkers from animal-origin protein profiles.	(Kritikou et al., 2022)
Czech	Cheese	Complete substitution with cheaper sorts of milk	27 kinds of cheese were analyzed with two mass spectrometric techniques–MALDI–TOF and LC–ESI–Q-TOF. The first technique could only partially distinguish cheeses fraud. However, LC–ESI–Q-TOF could successfully be used for analyzing samples. Sheep cheese of Dutch origin was estimated as the best cheese.	(Kuckova et al., 2019)
Italy	Cooked Buffalo Mozzarella	Fraudulent use of cow milk	Among all samples, one-third was almost pure cow Mozzarella cheese and 14 % of samples had less than 10 % cow milk. The European official method (based on different electrophoretic mobility in caseins) can effectively detect cow milk in buffalo Mozzarella.	(Locci et al., 2008)
Portugal	Goats’ milk cheese	Fraudulent presence of cow milk	The dPCR method was applied to assess cows’ and goats’ milk in cheese. Three among 17 commercial cheese samples were identified with fraudulent addition of cows’ milk (9–13 %).	(Mafra et al., 2007)
Italy	Buffalo Mozzarella	Adding frozen curd (FC)	TD-NMR experiments coupled with machine learning can be successfully used to detect common FFs.	(Mengucci et al., 2021)
Italy	Caprine and ovine	Fraudulent addition of bovine milk	A real-time PCR test was developed for detecting cow’s milk in caprine and ovine cheeses based on two target genes. Most of the 30 caprines and 51 ovine cheese samples were adulterated.	(Mininni et al., 2009)
Brazil	Butter cheese (BC)	Adulteration with soybean oil	FTIR-ATR spectroscopy coupled with multivariate analysis was used as a tool for detecting fraudulent addition of soybean oil (SO).	(Leite et al., 2019)
Croatia	Ewe and goat cheeses	Cow's milk	They determined cow γ2- and γ3-caseins from cheese after electrophoretic separation on urea-polyacrylamide gels from the homologous proteins of ewe or goat milk. The control of cheese adulterations has successfully been implemented.	(Spoljaric et al., 2013)
Greece	Packaged yellow cheeses, packaged white cheeses, PDO cheeses	Adulteration	The examination revealed that packaged yellow cheeses exhibited the lowest percentage of all the investigated categories, at 15 %. Conversely, the category of foods for pets exhibited the highest percentage of all the examined categories, measuring 54 %. The rates of mislabeling for various food categories stood at 26 % for milk, 29 % for packaged white cheeses, 26 % for PDO cheeses, 35 % for frozen fish products, and 34 % for processed meats, respectively.	(Stamatis et al., 2015)
Italy	Parmigiano Reggiano	Animal products authentication	The genetic test was successfully applied.	(Russo, Fontanesi, Scotti, Tazzoli, & Stefania Dall’Olio, and Roberta Davoli., 2007)
Spain	Ewes’ milk and cheese	Adulteration with goat’s milk	An indirect ELISA test could detect goats’ milk (1–25 %) in ewes’ milk and cheese using anti-goat casein antibodies.	(Rodriguez et al., 1991)
Italy	Buffalo mozzarella	Adulteration	A novel ultra-high performance LC-MS based on MRM was provided to monitor specific transitions of a novel species-specific proteotypic peptide marker derived from β-casein.	(Russo et al., 2012)
Italy	Water buffalo ricotta	Bovine's milk	MALDI-TOFMS was used successfully as a new tool for assessing adulterated buffalo ricotta with bovine milk.	(Russo, Rega, & Chambery, 2016)
Brazil	Buffalo mozzarella	Cow's milk	Adding cow milk to buffalo milk in preparation for mozzarella resulted in the modification of Short-chain fatty acids and cholesterol content, along with, nutritional indices. Cholesterol content was analyzed using a high-performance liquid chromatograph, and Short-chain fatty acids using Trace-GC-Ultra gas chromatography.	(Viana et al., 2020)
Argentina	Grated hard cheeses	Higher rate of additives than regulated and non-authentic materials used for increasing bulk and weight	NIR spectroscopy and multivariate analysis were applied to determine grated hard cheese. Two different food, including commercial grated cheese samples (starch content not declared on the label) and cheese-based foods with aggregates (starch content declared on the label) were assessed. NIR can perform well when detecting samples adulterated with starches.	(Visconti et al., 2020)
Argentina	Grated hard cheeses	Higher rate of additives	The image analysis had a high sensitivity (82 %) and could detect additive in samples.	(Visconti et al., 2023)
Italy	Grated hard cheeses	Different ingredient	*δ*^13^C, *δ*^2^H, *δ*^15^N, *δ*^34^S and Sr, Cu, Mo, Re, Na, U, Bi, Ni, Fe, Mn, Ga, Se, and Li were analyzed in the stable isotope ratio method and found 260 out of 264 samples have the correct classification.	(Camin et al., 2012)
Italy	Derived Mozzarella	Bovine's Casein	–	(Addeo, Pizzano, Nicolai, Caira, & Chianese, 2009)
Brazil	Buffalo cheese	Cow's milk	The sensory analysis was used for the detection of Adulteration. The sensory evaluations indicated that these differences were not perceptible by the consumers.	(Cardoso et al., 2019)
Iran	Sheep yogurt and cheese	Adulteration with cow or goat milk	Multiplex PCR assay was used to detect cow’s or goat’s milk in sheep products, considering the limit of detection of 2 % and 4 % in sheep yogurt and cheese, respectively. Only 20 % of the cheese samples and 27.5 % of the yogurt samples contained pure sheep milk.	(Zarei et al., 2016)
Slovak republic	Sheep cheese, milk, and bryndza	Adulteration with raw and heat-treated cow milk	Commercial ELISA was applied to detect adulteration of sheep milk products with cow milk. 9 samples of bryndza were found to have 11.56 % to 14.3 % cow milk. The ELISA tests can successfully identify cow milk presence, but they can’t assess its amount exactly due to irreversible changes caused during the manufacturing process.	(Zeleňáková et al. 2016)
Germany	Feta and Mozzarella	Rapid animal species identification	MALDI-TOF MS was successfully used to evaluate the source of two widely consumed cheeses, mozzarella and feta.	(Rau et al., 2020)
Italy	Buffalo Cheese	Cow's Milk	PCR was able to detect cow's milk in mozzarella labeled cheeses.	(Bottero, Tiziana Civera, Anastasio, & Turi, and Sergio Rosati., 2002)
Brazil	Grated parmesan cheese	Ripening time	Urea-PAGE electrophoresis could show that 80 % of the cheeses were ripe and 32 % had low fat.	(Fagnani, Damião, Trentin, & Zanoni, 2022)
Portugal	PDO	Cheaper/lower-quality milk	A Randomly Amplified Polymorphic DNA (RAPD) and Sequence Characterized Amplified Region (SCAR) were successfully used for the first time by combining these two methods to investigate cheese adulteration.	(Cunha et al., 2016)
Argentina	Hard-cheeses	Additives such as cellulose and silicon dioxide	Digital image analysis was able to detect fraud with an accuracy higher than 82 %. Adulteration included additives such as cellulose and silicon dioxide and foreign substances.	(Visconti et al., 2023)
Italy	Mozzarella	Bovine and water buffalo milk	Duplex-PCR was able to detect very low concentrations of DNA (1 pg).	(Rea et al., 2001)
Spain	Mozzarella	Cow's milk	The mitochondrial 12S ribosomal RNA gene was detected up to a threshold of 0.1 % by PCR method.	(López-Calleja et al., 2005)
USA	Halloumi	Milk's animal origin	Triplex-PCR was used to assess adulteration. The absence of adulteration in this type of cheese means that there is at least 51 % sheep or goat milk. This investigation showed that 2 out of 6 cheese samples were adulterated.	(Kastanos, Papaneophytou, Georgiou, & Demoliou, 2022)
France	Goats' cheeses	Cows' milk	PCR was able to detect cow's milk at a concentration of less than 0·1 % by identifying the mitochondrial sequence.	(Maudet & Taberlet, 2001)
Italy	*Stelvio* cheese	External components	Isotope ratio mass spectrometry (IRMS) was able to detect up to 6.5 % milk powder in the samples.	(Capici, Mimmo, Kerschbaumer, Cesco, & Scampicchio, 2015)
Portugal	Serra da Estrela	Milks from cheaper sources	Milk from Mocha sheep and cow that were mixed with Serra da Estrela were identified by PCR method.	(Silva, Baptista, Baptista, Teixeira, & Domingues, 2021)
Colombia	Buffalo mozzarella	Cow's milk	In 6 commercial cheeses examined by the RP-HPLC method, 4 cheeses were adulterated.	(Gonçalves et al., 2016)

IEF: Iso electro focusing, MBC: Mozzarella di Bufala Campana, WB: water buffalo, RP-LC–MS: RP liquid chromatography– tandem mass spectrometry, ddPCR: Droplet digital Polymerase Chain Reaction technique, PDO: Parmigiano Reggiano, QPCR: Quantitative real-time polymerase, FT-NIR: Fourier transform near-IR, PCR: Polymerase chain reaction, MALDI-TOF-MS: Matrix-assisted Laser Desorption/Ionization -Time-Of-Flight Mass Spectrometry RT-qPCR: Reverse transcription quantitative PCR, SDS-PAGE: Surfactant sodium dodecyl sulfate with polyacrylamide gel electrophoresis, ELISA: The enzyme-linked immunosorbent assay, SO: soybean oil, RAPD: Randomly Amplified Polymorphic DNA, SCAR: Sequence Characterized Amplified Region, IRMS: Isotope ratio mass spectrometry, RP-HPLC: Reverse-phase high-performance liquid chromatography.

### 2.4 Results

**Fig. 4:**
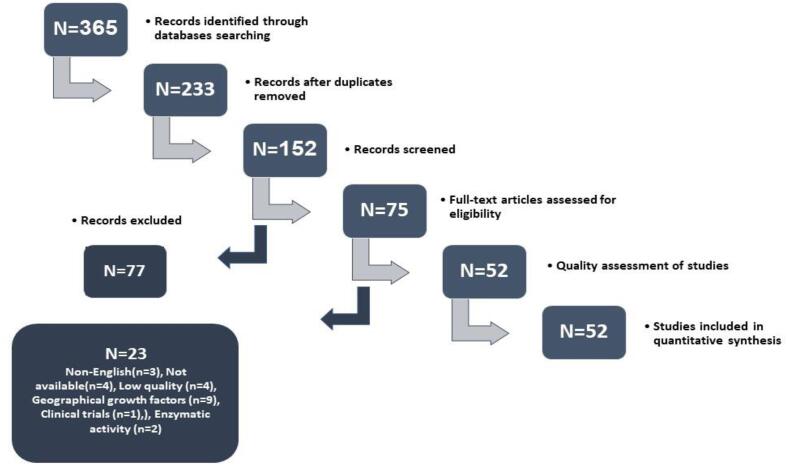
PRISMA checklist.

According to the search results, 365 articles were obtained in PubMed, web of science, Scopus, and Science direct database. With the removal of duplicate articles, 233 articles were selected for evaluation in the title and abstract sections, and inappropriate articles were removed for reasons (Investigation of livestock, geographical origin, non-English article, review, and chapter of book, not available, unrelated enzymatic activities and articles that did not pay attention to fraud). At this stage, all 75 articles were reviewed. 52 articles were selected by two people after checking the quality of the articles. This study was conducted based on the PRISMA checklist, and is shown in Fig. 4.

### 2.5 The descriptive results of the screened manuscript

52 articles were evaluated for this article. The name of the first author, year of publication, country, type of cheese, type of fraud, and evaluation, including the study method and the result of the study, was written in this Table 1.

## 3 Methods of detecting fraud in cheese

### 3.1 Stable isotope

The isotopic ratio refers to the atomic abundance ratio of two isotopes of a related element, for example, O and Nd, including 18O/16O or 143Nd/144Nd. The advantage of using ratios instead of absolute abundance of a particular nuclide is better accuracy and efficiency. The comparison of the 143Nd and 144Nd signals can be done at the ppm (1 part per million) level of accuracy, which results are two to three orders of magnitude more accurate than counting each nuclide individually (Albarede, 2011). These ratios can be related to specific regions because studies show that environmental isotopic ratios form specific patterns. These characteristics and proportions can help to measure the authenticity of foods and identify their production areas (O'Sullivan, Schmidt, & Monahan, 2022). For example, milk production at an altitude of 1100 m and 200 m can be identified by the isotopic ratios of O and H for higher altitudes and the isotopic ratios of H, O, N, and S for lower altitudes. Another thing that affects the ratios is food fortification. For example, milk enrichments differed significantly between sites for both 18O and 2H (Kalpage et al., 2022). Examination of cheeses shows that isotopic ratios between 13C/12C and 15N/14N of casein can be a useful approach to evaluate their place of origin. For example, these ratios have been used in the evaluation of European Emmental cheeses. Examination of *δ*^13^C, *δ*^15^N, *δ*^2^H, *δ*^87^Sr-values was able to successfully separate cheeses from different regions, including Finland, Savoie, and Bretagne. The results of various studies show that stable isotope ratios can be used to check the geographical location and authenticity of cheese (Karoui & De Baerdemaeker, 2007).

The international standard for stable isotope analysis is represented by the following formula, where R represents the ratio between heavy and light isotopes:

$$\delta ‱=\left(R_{\mathrm{Sample}}-R_{\mathrm{Standard}}\right)/R_{\mathrm{Standard}}*1000$$

One of the factors that can affect isotope ratios is the production process. The production process in cheese includes coagulation, the thermal process above 50 ◦C, acidification, salt, and removal of fat, especially glycerol. Bontempo, Lombardi, Paoletti, Ziller, and Camin (2012) reported that different processes and technologies that can lead to cheese production can cause possible changes in stable isotope ratios. They observed that cheese-making was able to create a different isotopic ratio. For example, Mozzarella di Bufala Campana PDO milk and cheese reported *δ*^2^H and *δ*^18^O, respectively (Bontempo et al., 2012).

Manca et al. (2006) investigated the relationship between isotopic ratios and geographical origin in Peretta Cows' Milk Cheese. Their study aimed to identify the original cheese among cheeses sold with the same name but with different ingredients. They examined 3 groups, including cheeses made with imported ingredients, cheeses made with intensive agricultural ingredients, and cheeses made using milk from free-range or pasture-grazed cows in Sardinia. Their investigated isotopic ratios include 13C/12C, 15N/14N, D/H, 34S/32S, and 18O/16O. Determination of the isotopic data *δ*^13^C, *δ*^15^N, *δ*^2^H, and *δ*^34^S was performed in the casein fraction, whereas *δ*^18^O and *δ*^13^C were determined in the glycerol fraction. Examining the results showed that the biggest differences in cheeses produced with different ingredients were observed in 13C/12C, 34S/32S, and 18O/16O. The results of their investigation showed that the isotopic profile of factory products was similar to cheeses produced from imported raw materials (Manca et al., 2006).

### 3.2 Near-infrared (NIR)

The selective interaction of infrared rays with food molecules can be measured using spectroscopic analyses, including NIR. The high diversity of cheeses and the presence of complex matrices increase the challenges of using NIR to assess cheese adulteration. Another challenge of NIR is the initial lack of ability to differentiate the geographical origin of milk, but overall, this method has been able to detect the ripening time of cheese, chemical composition, and manufacturing technique. In studies from 850 to 1048 nm wavelength region has been used to evaluate the adulteration of cheeses. (Cardin et al., 2022).

Visconti et al. (2020) investigated adulteration in commercial grated cheese samples. The purpose of their investigation was to evaluate additives such as starch, cellulose, and bulking agents because the addition of starch can reduce the quality and functional characteristics of such products. Two different foods, including commercial grated cheese samples (starch content not declared on the label) and cheese-based foods with aggregates (starch content declared on the label), were assessed. Excellent classification results were obtained with partial least squares discriminant analysis (PLS-DA) as adulterated samples were discriminated from original (unadulterated) samples. They concluded that NIR can detect starch in grated cheese as an affordable and fast technology (Visconti et al., 2020).

Dvorak, Mlcek, and Sustova (2016) compared two methods of ELISA and FT-NIR to evaluate adulteration in goat cheeses. Their investigation method was partial least-squares (PLS) model. The findings of the study indicate that the FT-NIR technique outperformed the ELISA method in detecting cow's milk mixture in goat cheese, with sensitivity to concentrations as low as 1 %. Moreover, it was revealed that the ELISA method was noted to be less accurate and less satisfactory than the FT-NIR method (Dvorak et al., 2016).

### 3.3 Image analysis

New methods of controlling and monitoring the safety of food products have expanded significantly with recent developments. For example, image analysis and the resulting data can make food product safety checks more accurate and faster. The applications of image analysis technology are in agriculture, food production, biomedicine, food transportation, and sales. These techniques include image segmentation, defect segmentation, feature extraction, training, and classification (Kurade et al., 2023).

By expanding the relationship between artificial intelligence and machine learning in the food industry, the use of novel techniques such as image recognition techniques can help classify food products to predict food spoilage, quality, and authenticity assessment. For example, this technique can report corruption and adulteration of food products much faster than conventional methods using image data (Khan, Kumar, Dhingra, & Bhati, 2021). Classification of products using artificial intelligence techniques such as SVM, KNN, J48, and RF in a computer vision system makes it easier to evaluate food product fraud. Other applications in safety assessment include the use of dynamic and targeted monitoring based on BN, which is used in food supply chain monitoring, Swarm intelligence (SI) in fresh food distribution, and SVM to check the safety of food products in transportation are some of the advances in the use of artificial intelligence in food products (Kurade et al., 2023). Also, reliable sources of novel data streams, which are a subset of machine learning, are effective in increasing the efficiency of these methods, which include text data, transactional data, and trade data. These sources can be used in the evaluation of foodborne illnesses, microbial contamination, and chemical, physical, and microbiological hazards in food products (Wang et al., 2022, Abedini et al., 2022).

Visconti, Vargas, Rodríguez, Di Anibal, and Delrieux (2023) investigated adulteration in grated hard cheeses. Their chosen method was an analysis of data obtained from image analysis. Grated cheese is one of the most prone cheeses to use invalid food additives that can significantly affect the characteristics of the product quality. Food additives include starches, flour, cellulose, silicon dioxide, wheat-semolina, and sawdust. The authors used the method of multivariate classification analysis and color histograms (obtained from digital images). After analyzing the results, the method had a high sensitivity of 82 % and was able to detect adulterated samples. They concluded that the use of image analysis methods could be one of the fast and accurate methods to evaluate adulteration in grated hard cheeses (Visconti et al., 2023).

### 3.4 PCR-based methods

PCR is a method that contributes DNA to identify different species. The main reason is DNA thermal stability in comparison with other compounds like lipids and proteins and it remains after manufacturing processes. In the food industry, several PCR-based methods are designed and used for detecting fraud. Among other analytical techniques, PCR-based methods are reported to be the fastest and the most accurate diagnostic approach (Quan et al., 2018, Waters and Shapter, 2014). In recent years, single or multiplex PCR approaches have also been informed for authenticity confirmation of cheeses produced from the milk of sheep, goat, buffalo, or their mixture (Baptista et al., 2021, Rentsch et al., 2013).

Zarei, Maktabi, Yousefvand, and Tajbakhsh (2016) evaluated 40 cheese samples in Iran, 20 % of which contained pure sheep milk in the context of molecular assay utilizing multiplex PCR, the threshold of detectability for cow or goat milk content in sheep cheese was determined to be 4 %.

The analyzation of cheese samples revealed the existence of goat's and cow's milk adulterants in 17.5 % and 35 % of the samples, correspondingly. Multiplex PCR is a useful and straightforward method for the detection of low contents of goat’s or cow’s milk in sheep milk products (Zarei et al., 2016). A technique involving real-time polymerase chain reaction (PCR) was utilized to detect and assess the presence of bovine milk within MBC cheese (Cutarelli et al., 2021) and in ovine milk (Guo et al., 2020). PCR was devised to identify and quantify cow's milk in caprine and ovine cheeses using two specific target genes. In pursuit of the aforementioned objective, a total of thirty Caprine cheese samples and fifty-one Ovine cheese samples were subjected to analytical evaluation. The outcomes illustrated that the majority of the samples were found to be adulterated with bovine milk (Mininni et al., 2009). Klancnik et al. (2016) used quantitative reverse transcription PCR (RT-qPCR) to investigate the adulteration of 46 cheese samples. The research findings indicate that 5 % of the goat cheeses and 12 % of the sheep cheeses were found to possess a cow's milk content exceeding 5 %. Cheeses containing detectable content of bovine milk might have a health risk to people allergic to bovine milk (Klančnik, Toplak, Kovač, Ogrinc, & Jeršek, 2016). Potential allergens like cow milk casein protein may have fatal results (Kuckova, Zitkova, Novotny, & Smirnova, 2019).

Mafra, Roxo, Ferreira, Beatriz, and Oliveira (2007) reported that the duplex PCR can detect goats’ and cows’ milk simultaneously in cheese. Out of 17 commercial cheeses, 3 samples were identified with fraudulent addition of cows’ milk (9–13 %). This method can measure 0.1 % of bovine milk in caprine milk cheese through a 35-cycle duplex polymerase chain reaction and identify cheese adulteration within the 1–60 % range using a 30-cycle duplex PCR. According to the information provided, two varieties of cheese containing mixed milk did not indicate the presence of goat's milk on their labeling (Mafra et al., 2007). The accuracy of this method was >other measurements by PCR technique (0.5 %) in detecting bovine milk adulteration (Feligini et al., 2005, Golinelli et al., 2014). A recent scholarly investigation conducted by Guo et al. (2019) revealed the efficacy of a newly developed triplex TaqMan real-time PCR technique as a highly precise and sensitive tool for detecting fraudulent representation of cow and/or goat milk within cheese products (Guo et al., 2019). *Taq*Man real-time PCR is more effective and accurate than real-time PCR using a fluorescent dye (Guo et al., 2019, López-Calleja et al., 2013). Also, ddPCR due to its high sensitivity was recommended as a straightforward approach for detecting DNA of buffalo and/or cow milk in PDO buffalo mozzarella cheese.

The aforementioned approach exhibits a remarkable level of precision in gauging diminished concentrations of bovine lacteal secretion. However, its utilization entails a considerable investment of time and personnel resources, coupled with a restricted scope of application in the context of customary milk screening endeavors for dairy commodities (Kuckova et al., 2019).

### 3.5 Spectrometry-based methods

Among all strategies, mass spectrometric analysis is a common method used for studying principal components of cheeses, such as fatty acids and proteins. Mass spectrometric techniques help analyze the protein composition of cheese samples, which differs based on the milk source. Whole proteins or peptides of each sample are digested enzymatically and analyzed for detecting fraud (Abdol-Samad et al., 2021, Kuckova et al., 2019). MALDI–TOF MS and LC coupled with electrospray ionization and quadrupole time-of-flight MS (LC–ESI–Q-TOF MS) methods are based on enzymatic cleavage of proteins (caseins) into peptides which are detected and compared with standards.

The verification process of 27 types of cheese utilizing two distinct techniques has indicated that the quantity of bovine α-s1 and α-s2-specific casein sequences identified in the analyzed samples exceed that of the sheep sample sequences. This outcome suggests a notable imbalance in the composition of the protein components within the compared samples. The presence of bovine casein can result in the contamination of cheese through its manufacture. The present study has revealed that the outcomes obtained through the use of MALDI-TOF may not be considered appropriate for the authentic identification of various types of cheeses. This could potentially be attributed to the incorporation of additives or the incomplete homogenization of goat's or sheep's milk and bovine milk.

Higher quality outcomes have been obtained by LC–ESI–Q-TOF technique and, it seems to be a more suitable technique for the research (Kuckova et al., 2019), as shown in Table 1. Furthermore, one study found that an alternative approach can be applied for detecting animal species in mozzarella and feta cheeses via MALDI-TOF-MS. They compared peptide profiles of cheese samples from sheep, buffalo, goat, and cow’s milk in their study. This method can be used to classify all kinds of cheeses. In addition, this method, along with chemometrics, has been used to investigate cheese fraud. One example is the study conducted by Kritikou et al. (2022), in their study employed MALDI-TOF technology in conjunction with chemometrics (biomarkers) to differentiate feta cheese from other white cheese varieties and to identify contamination with bovine milk in a sample dataset comprising 91 samples. The combination of the above methods was known as a reliable approach for the detection of Protected Designation of Origin (PDO) in feta cheese authenticity for routine analysis (Kritikou et al., 2022). Russo et al. (2012) successfully used ultra-high-performance LC/MS based on MRM acquisition mode for the authentication of buffalo mozzarella cheese. This new and accurate method is very useful in evaluating fraud by using the phosphorylated β-casein f33-48 tryptic peptide, which is known as a new species-specific prototypic marker (Russo et al., 2012). LC/ESI-MS is another method based on analyzing two peptides of sheep (casein), which can detect sheep's milk in dairy products such as cow and goat cheeses with 2 % accuracy (Guarino et al., 2010, Valletta et al., 2021).

According to the results of the mentioned studies, spectrometric-based analyses are applicable and offer a suitable methodology, which is based on analyzing specific peptides or peptide sequences, both of which differ in milk and cheese samples according to their sources. Furthermore, a combination of spectrometric-based with (bio-)markers seems to be an efficient way of detecting fraud in cheese types like feta.

### 3.6 Electrophoretic methods

Classic electrophoretic methods, including IEF are recognized as cheap, fast, and easy to apply. IEF separates proteins based on Isoelectric Point (IP), which differs among various proteins (Masci et al., 2022). In the dairy industry, IEF methods can be used for exploring milk sources, also coupled with other techniques like HPLC. All of these methods work based on protein physicochemical properties (Chien et al., 2022). The proposed method is faster, easier to perform, and doesn’t require neurotoxic polyacrylamide gels unlike the method, which is currently used and is based on IEF analysis (Guarino et al., 2010). Based on the studies, IEF of γ-caseins after hydrolysis by plasmin is a reference technique for sheep, goat, and buffalo dairy products, adulterated with cow milk. In a study, such a technique was applied, based on the extraction and purification of denatured whey proteins, and separation via IEF. The results showed that the accuracy of this method was effective for measuring 5 % of cow whey in buffalo whey in ricotta samples (Fuselli et al., 2015).

The IEF method on γ2- and γ3-casein derived from the hydrolysis of β-casein can be used for the detection of raw and heat-treated cow milk in ripened and fresh cheeses made from goat or ewe milk, or admixture of them (Spoljaric et al., 2013). Caira and her colleagues in 2019, an enhanced version of the IEF technique was developed to evaluate the characteristics of WB milk, which serves as a crucial ingredient in the production of Mozzarella di Bufala Campana (MBC) cheese. The examination of casein patterns in whole bovine milk originating from both domestic and international sources facilitated the aforementioned findings. Through a series of methodological approaches including IEF, mass spectrometry MS, and immunoblotting assays, the researchers successfully detected specific proteotypic peptides derived from the parent proteins of αs1-casein and β-casein within Western Blot (WB) milk. This analytical identification method proved effective in differentiating Italian from foreign WB milk samples (Caira et al., 2019).

Among all protein-based methods developed for detecting the addition of milk from different species, electrophoresis is notable due to its low cost. Using polyacrylamide gel electrophoresis (PAGE) in the presence of the surfactant sodium dodecyl sulfate (SDS) is the most common way of this technique. In case of similarity between the protein profile of the milk of some species, evaluation of water-soluble peptides (WSP) is essential (Gonçalves, de Jesus Silva, & Conceição, 2017). Gonçalves and colleagues. In 2017, an evaluation was conducted on the quality of buffalo milk mozzarella utilizing electrophoresis (SDS-PAGE) as a means of detecting the presence of cow milk within the samples. Eighteen distinct cheese samples were produced through the combination of buffalo milk with varying proportions (2.5 %, 5.0 %, 10 %, 20 %, 30 %, 40 %, or 50 %) of cow's milk. The samples were subsequently subjected to frozen storage either immediately after production or after 20 days. WSP and proteins were analyzed using SDS-PAGE under denaturing conditions. They reported 28 % of the commercial samples contained cow’s milk (Gonçalves et al., 2017).

The fraudulent inclusion of bovine milk in buffalo mozzarella was brought to light through the employment of SDS-PAGE for the total protein and peptide separation of bovine and buffalo milk. Of the 18 commercial samples of buffalo mozzarella analyzed, it was discovered that five had been supplemented with cow's milk. The electrophoretic analysis of protein fractions is constrained by the resemblance of the protein profile among certain species in milk (Gonçalves et al., 2017). Electrophoresis is a highly noteworthy technique, distinguished by its ability to provide favorable results among a multitude of other methodologies. Furthermore, this method has been recognized for its precision in detecting instances of cow's milk adulteration in samples. Nonetheless, it does not exhibit a high degree of efficacy and the detection process is characterized by a notable time requirement (Caira et al., 2019). The main drawbacks of protein-based approaches are uncertain results that can be obtained in cooked and half-cooked curds along with minimal contamination and interpretation problems because of the overlapping of species-specific bands that may need integration with hard immunoblotting steps (Di Domenico, Di Giuseppe, Wicochea Rodríguez, & Cammà, 2017). Moreover, the prolonged duration of manufacturing processes and the considerable thermodynamic instability of proteins when subjected to temperatures exceeding 40 °C are inherent limitations of utilizing protein-based methodologies (Sezer, Bjelak, Velioglu, & Boyaci, 2022). According to the results of several studies, these protein-based techniques are useful approaches for figuring out fraudulent addition of different species to cheeses like Mozzarella di Bufala Campana.

### 3.7 The enzyme-linked immunosorbent assay (ELISA)

The ELISA is one of the widely used methods for measuring food components that can act as antigens. This immunoassay is based on an interaction between an antibody and an antigen, one of which carries a covalently bound enzyme.

The aforementioned enzyme effectively catalyzes the chemical transformation of a substrate into a chromatic end-product. These steps can be done using fluorescence detection or spectrophotometry (Dai et al., 2022). The fundamental principle underlying the operation of the Enzyme-Linked Immunosorbent Assay (ELISA) lies in its ability to detect potential adulteration via the interaction of monoclonal or polyclonal antibodies with various constituents such as bovine immunoglobulins, whey proteins, and casein-macro peptide (CMP). It’s possible to analyze multiple samples in a single run (Masci et al., 2022). ELISA is commonly applied in the form of an immunoassay for the analysis of milk, which has the benefits of low cost, highly sensitive, rapid, and easy to use (Yildirim-Tirgil, 2022). Zeleňáková and colleagues in 2016, investigated by ELISA the detection of both raw and heat-treated cow milk in cheese, sheep milk, and commercially available “Bryndza”. They reported that ELISA was capable of detecting cow's milk. But irreversible changes in the production process have detrimental effects on the accuracy of identification.

It is suggested that to evaluate cheese adulteration, the procedures should be carefully examined and sampled (Zeleňáková, Židek, Čanigová, Žiarovská, Zajác, Maršálková, Fikselová, & Golian, 2016).

The utilization of commercialized ELISA products has also been observed in various research studies. To date, this particular product has been utilized in assessing the presence of adulterants in cheese made from cow's milk and milk derived from sheep (Asensio, Alonso, Garcia, & Martín, 2008). In another study, Hurley, Coleman, Elyse Ireland, and Williams (2004) investigated adulteration in goat soft cheeses using ELISA. They reported that ELISA has a high ability to detect adulteration and detected 0.01 % admixture of cow's milk in goat soft cheeses. In addition, they stated that detecting undamaged proteins can be difficult for this method (Hurley et al., 2004). Choosing the combination of tests with each other and the correct choice of the required ELISA method is the key point in the evaluation of fraud in milk and cheese (Zeleňáková et al. 2016).

The common adulteration of the presence of cow's milk in cheese types such as Pez can be evaluated using many techniques. Immunological and molecular-biological methods are among the techniques used in this field. Cow milk admixtures can be identified from 0.01 to 5 % via these techniques (Dvorak et al., 2016). Leite et al. (2019) find out that were approximately similar to the previous outcomes, which demonstrated Fourier-transform infrared spectroscopy - Attenuated Total Reflection (FTIR-ATR) allied with chemometrics can be used as a great and rapid analytical method in the authenticity of butter cheeses. A partial least squares technique was capable detection of soybean oil in butter cheeses in a small amount (Leite et al., 2019). It is worth mentioning that ELISA is capable of identifying and quantifying the amount of adulteration in different cheeses, goat cheese for instance. According to the results of several studies, choosing the right methodology is essential for the correct investigation of milk and cheese samples.

### 3.8 Sensors

Sensors are one of the newly emerged methods for detecting adulteration which are divided into three groups based on their transducer; electrochemical, piezoelectric, and optical. In some cases, they are also coupled with MS, IR spectroscopy, or IEF (Alizadeh Sani et al., 2023). This method focuses mostly on the milk protein fraction (Hebling e Tavares, da Silva Medeiros, & Barbin, 2022). Based on earlier research, the introduction of bovine milk into mozzarella cheese modulates its lipid fraction and alters its fat profile. This itself is considered a clear fraud. Fatty acids such as C4:0, C16:0, C22:0, and C16:1 declined, while C8:0 and C10:0 increased. With the inclusion of cow milk to buffalo milk into mozzarella processing, the parentage of short-chain saturated fatty acids changed. Also, the level of cholesterol increased. Compared to cow milk, buffalo milk contains less cholesterol (Viana et al., 2020). Checking the profile of fat and fatty acids can be done using techniques such as chromatographic methods. But one of the disadvantages of these methods is the high time they require (Oteri et al., 2022).

A novel study unveiled the development of a speedy and remarkably sensitive silicon photonic sensor used to precisely identify the adulteration of feta and buffalo mozzarella cheese with bovine milk. Specifically, a domestic anti-bovine κ-casein rabbit antiserum was utilized for this purpose.

The sensor in question was identified as an impressive economic instrument with a brief trial period and a minimal limit of detection. As an example, fraudulent activity was detected in mozzarella and feta cheeses at rates of 0.5 % and 0.25 % (w/w), respectively. These percentages were found to be below the acceptable limit of 1 % (w/w) set by the European Commission concerning the presence of bovine milk in cheeses made from the milk of other bovine species. The milk substitution legal threshold ought to be reduced to 0.99 %, while it is worth noting that alimentary fraud occurs when the substitution rate equals or exceeds 1 % (Angelopoulou et al., 2021). Sensors are optimized as one of the novel methods for fraud identification due to their accuracy and quickness. Also worth mentioning is that there are several types of sensors with different detection factors, specific fatty acids in buffalo mozzarella or feta cheese.

### 3.9 Sensory analysis

Sensory evaluation is a technique that uses human sensory reactions to detect off-flavors, predict the acceptability of a product, etc. (Hebling e Tavares et al., 2022). In contrast to other methods, the sensory analysis doesn’t have a specific factor, does not require specific equipment or highly qualified personnel, and becomes more reliable with trained panelists. Despite other analytical techniques being used for identifying adulteration, it is of high value to figure out at which point consumers detect adulteration (Golinelli et al., 2014). Also in many countries, Brazil, for example, there isn’t any reference method for testing mislabeling or adulteration (Aquino et al., 2014). Although sensory evaluation has been discussed in many studies, its application for detecting fraud in cheese is still new.

Cardoso et al. (2019) assessed sensorial consumers’ acceptance of fraudulent cheeses. In this study willingness to buy and sensory factors of cheese samples were experimented with along with their physicochemical factors like moisture. They concluded that cheeses made from an admixture of cow and buffalo milk are acceptable although there are physicochemical differences. In their study, 30 to 60 untrained judges tested cheese samples made from different milk percentages. Units were made of 100 % buffalo milk, 50 % buffalo milk, and 50 % cow milk, or 100 % cow milk and each batch of cheese contain 5 units. Judges could rate the color, flavor, and texture of the samples with a five-point hedonic ranking. According to their results, 59 % of testers liked the texture very much and 57 % of them liked the color very much (Cardoso et al., 2019). The results of organoleptic and sensory analyzes are generally reviewed in the opinion of consumers, experts, producers, and consumers.

### 3.10 Nuclear magnetic resonance (NMR)

With the increasing attention of consumers to food safety and wellness, modern methods are being improved and used. Time-Domain (TD)-NMR is recognized as a promising method for controlling food quality, one of which is experimenting authenticity of dairy products. In this method, the proton relaxation (transversal relaxation, T2) is monitored and provides information about the mobility of the nuclei, also unique information about bound water, free water, and an exchange between these two states (Qu & Jin, 2022). Mengucci et al. (2021) investigated the adulteration of Mozzarella di Bufala Campana, an Italian cheese with frozen curd that during the production process is known as a frequent fraud, and can’t be easily recognized by general analyzing methods, mainly because they focus on chemical composition. Using this newly improved technique, dehydration of caseins in cheese samples that were affected by frozen and refrigerated storage could be detected. In this experiment, researchers made four groups of samples from “Mozzarella di Bufala Campana” containing 0, 15, 30, and 50 % FC. They concluded that TD-NMR coupled with machine learning can be useful for fraud identification, especially when the structural level is altered and not the chemical composition (Mengucci et al., 2021). After all, due to its advantages like simple and fast measurement procedures and instrumental stability, this method has been investigated in the past few years and declared to be a useful method for controlling food quality and identifying fraud.

## 4 Future perspective

The methods used to evaluate adulteration in cheese include PCR, spectrometry, ELISA, sensors, and NIR. But new methods such as artificial intelligence (AI) and machine learning are attractive topics for fraud evaluation. AI has a high ability to evaluate fraud with high accuracy. The methods based on AI include examining data through media (text mining, network analysis). AI can predict fraud and quality through a detailed examination of data and past studies (word embedding). Detailed examination of safety parameters through satellite and mobile photos (deep learning) is another emerging technique (Huck et al., 2016, Jahanbakhshi et al., 2021). For example, we can mention the European Media Monitor (EMM), which investigates FF by collecting detailed information. Many limitations have caused the use of AI in the examination of cheese and food products to be delayed. Limitations such as the trained workforce delayed transfer of new technologies in the field of AI and the connection of AI with society and its explanation in the field of fraud investigation and food safety. There are many examples of the impact of AI on food safety. For example, checking melamine in milk powder with a mobile phone or checking contaminants of agricultural products with satellite imaging. It seems that due to the high capabilities of AI in detection, this technology is one of the most attractive global trends for evaluating the fraud of dairy and food products (Rezazade et al., 2022, Schwarzinger, 2022).

Another thing of interest for cheeses is the removal of some stages of their production, which is a form of FF. For example, in PDO cheeses, stage removal and delay have been observed (Like the ripening period). AI and new photography methods will be interesting studies to investigate this issue in the future (Hebling e Tavares et al., 2022, Zhou et al., 2022). Also, with the expansion of demand for organic products, many parameters can be examined. For example, investigating the type of animal nutrition and the effect on dairy products can be another subject investigated with AI. Certainly, future studies will focus on increasing focus, miniaturizing fraud assessment devices, and reducing time in fraud assessment (Soon, 2022, Iymen et al., 2020, Sadighara et al., 2022).

## 5 Conclusion

This systematic study investigated the types of fraud in cheese and their identification methods. Fraud in the food industry can affect human health with economic losses. In cheese, the most common frauds were reported, including using other milk or mixing milk. It was also observed that vegetable oils such as soybean oil were used to modify the fat profile in cheese. In addition, other materials such as curd and starch are also among the materials that can be used in the adulteration of cheeses. It seems that some food additives can be used to adulterate cheese, which researchers are suggested to investigate in future studies. These additives sometimes make food products toxic and reduce their nutritional value. It should be noted that people have no knowledge of fraud and cannot evaluate it at home or with simple tools. This issue indicates that authorities responsible for evaluating FF and quality should inform and raise awareness more effectively. Inspection in this field needs to be reformed and trained because the high volume of fraud in food products in the world shows that this issue is one of the concerns for health and the economy. The methods of checking cheese fraud included PCR, stable isotope, image analysis, NIR, spectrometry, electrophoretic, ELISA, sensors, sensory analysis, and NMR; the methods based on PCR and spectrometry were the most used in checking cheese fraud, respectively. The sensory analysis was also reported to be the least used. The highest amount of fraud was observed in Mozzarella, Ricotta, hard, feta, butter, and Parmigiano Reggiano cheeses, respectively (Animal; goat, Caprine, and sheep, respectively). The review of the articles showed that one of the main types of fraud in the dairy industry, especially cheese, is the use of a mixture of milk to produce products and adulteration. Due to its high price and high uses, Mozzarella cheese had the highest number of counterfeits among all cheeses. In addition, the study of countries showed that the highest publication for adulterated cheese was from Italy, Brazil, and Greece.

## Declaration of Competing Interest

The authors declare that they have no known competing financial interests or personal relationships that could have appeared to influence the work reported in this paper.

## Data Availability

The data that has been used is confidential.
